# (*E*)-*N*′-(5-Bromo-2-meth­oxy­benzyl­idene)-2-chloro­benzohydrazide

**DOI:** 10.1107/S1600536811034623

**Published:** 2011-08-27

**Authors:** Xiao-Yan Li

**Affiliations:** a Zibo Vocational Institute, Zibo 255314, People’s Republic of China

## Abstract

In the title compound, C_15_H_12_BrClN_2_O_2_, the dihedral angle between the two substituted aromatic rings is 77.8 (3)°. The mol­ecule exists in a *trans* conformation with respect to the methyl­idene unit. In the crystal structure, inversion dimers linked by pairs of N—H⋯O hydrogen bonds generate *R*_2_^8^(8) loops.

## Related literature

For the crystal structures of some related hydrazone compounds, see: Li (2011*a* ▶,*b* ▶); Hashemian *et al.* (2011 ▶); Lei (2011 ▶); Shalash *et al.* (2010 ▶). For hydrogen-bond notation, see: Bernstein *et al.* (1995 ▶).
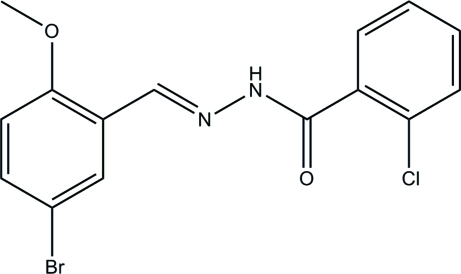


## Experimental

### 

#### Crystal data

C_15_H_12_BrClN_2_O_2_
*M* *_r_* = 367.63
Monoclinic, 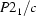

*a* = 11.312 (2) Å
*b* = 7.374 (2) Å
*c* = 17.979 (3) Å
β = 91.972 (3)°
*V* = 1499.0 (5) Å^3^
*Z* = 4
Mo *K*α radiation
μ = 2.93 mm^−1^
*T* = 298 K
0.12 × 0.10 × 0.07 mm

#### Data collection

Bruker SMART CCD diffractometer
Absorption correction: multi-scan (*SADABS*; Sheldrick, 1996 ▶) *T*_min_ = 0.720, *T*_max_ = 0.821
5249 measured reflections
2451 independent reflections
1877 reflections with *I* > 2σ(*I*)
*R*_int_ = 0.050

#### Refinement

*R*[*F* ^2^ > 2σ(*F* ^2^)] = 0.036
*wR*(*F* ^2^) = 0.094
*S* = 1.04
2451 reflections
194 parameters
1 restraint
H atoms treated by a mixture of independent and constrained refinement
Δρ_max_ = 0.46 e Å^−3^
Δρ_min_ = −0.46 e Å^−3^

### 

Data collection: *SMART* (Bruker, 1998 ▶); cell refinement: *SAINT* (Bruker, 1998 ▶); data reduction: *SAINT*; program(s) used to solve structure: *SHELXS97* (Sheldrick, 2008 ▶); program(s) used to refine structure: *SHELXL97* (Sheldrick, 2008 ▶); molecular graphics: *SHELXTL* (Sheldrick, 2008 ▶); software used to prepare material for publication: *SHELXTL*.

## Supplementary Material

Crystal structure: contains datablock(s) global, I. DOI: 10.1107/S1600536811034623/hb6337sup1.cif

Structure factors: contains datablock(s) I. DOI: 10.1107/S1600536811034623/hb6337Isup2.hkl

Additional supplementary materials:  crystallographic information; 3D view; checkCIF report

## Figures and Tables

**Table 1:** Hydrogen-bond geometry (Å, °)

*D*—H⋯*A*	*D*—H	H⋯*A*	*D*⋯*A*	*D*—H⋯*A*
N2—H2⋯O2^i^	0.89 (1)	1.99 (1)	2.882 (4)	175 (4)

Symmetry code: (i) 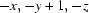.

**Fig. 1.**
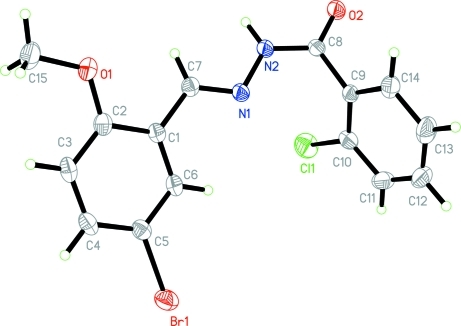
The molecular structure of (I) at 30% probability level. Hydrogen bonds are indicated by dashed lines.

**Fig. 2.**
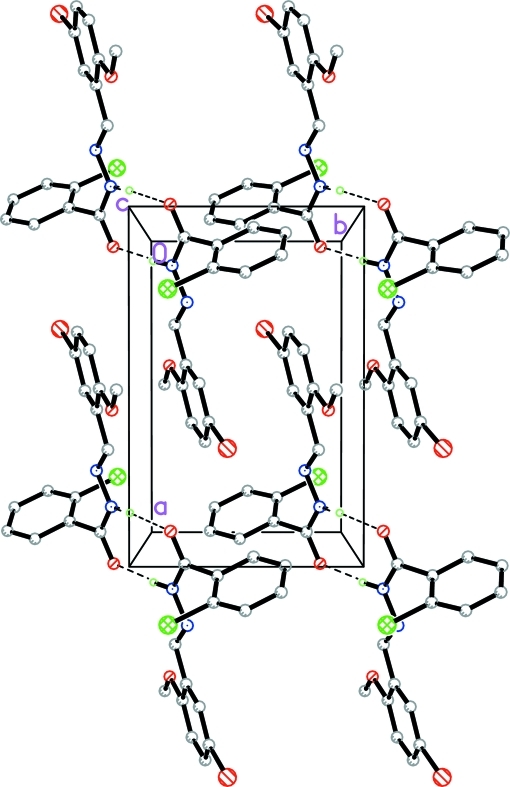
The packing of (I), viewed along the c axis. Intermolecular hydrogen bonds are drawn as dashed lines.

## supplementary crystallographic information

### Comment

In the last few years, hydrazones have been attracted much attention for their crystal structures (e.g. Li, 2011*a*; Hashemian *et al.*, 2011; Lei, 2011; Shalash *et al.*, 2010). The author reports herein the crystal structure of the title new hydrazone compound, (I).

In the title compound, Fig. 1, the dihedral angle between the two substituted aromatic rings is 77.8 (3)°. The molecule exists in a *trans* configuration with respect to the methylidene unit. The bond values are comparable to those observed in a similar compound the author reported recently (Li, 2011*b*). In the crystal structure, adjacent two molecules are linked through two intermolecular N—H···O hydrogen bonds (Table 1), forming a dimer (Fig. 2).

### Experimental

A mixture of 2-chlorobenzhydrazide (0.171 g, 1 mmol) and 5-bromo-2-methoxybenzaldehyde (0.215 g, 1 mmol) in 30 ml of ethanol containing few drops of acetic acid was refluxed for about 1 h. On cooling to room temperature, a solid precipitate was formed. The solid was filtered and then recrystallized from methanol. Colorless blocks of (I) were obtained by slow evaporation of the solution.

### Refinement

The N-bound H atom was located from a difference Fourier map and refined isotropically. The rest of H atoms were positioned geometrically [C—H = 0.93 and 0.96 Å] and refined using a riding model [U_iso_(H) = 1.2U_eq_(C) and 1.5U_eq_(C15)]. A rotating-group model was applied for the methyl group.

### Crystal data

**Table:**

C_15_H_12_BrClN_2_O_2_	*F*(000) = 736
*M**_r_* = 367.63	*D*_x_ = 1.629 Mg m^−^^3^
Monoclinic, *P*2_1_/*c*	Mo *K*α radiation, λ = 0.71073 Å
*a* = 11.312 (2) Å	Cell parameters from 1972 reflections
*b* = 7.374 (2) Å	θ = 3.1–24.9°
*c* = 17.979 (3) Å	µ = 2.93 mm^−^^1^
β = 91.972 (3)°	*T* = 298 K
*V* = 1499.0 (5) Å^3^	Block, colourless
*Z* = 4	0.12 × 0.10 × 0.07 mm

### Data collection

**Table:**

Bruker SMART CCD diffractometer	2451 independent reflections
Radiation source: fine-focus sealed tube	1877 reflections with *I* > 2σ(*I*)
graphite	*R*_int_ = 0.050
ω scans	θ_max_ = 25.0°, θ_min_ = 2.9°
Absorption correction: multi-scan (*SADABS*; Sheldrick, 1996)	*h* = −13→13
*T*_min_ = 0.720, *T*_max_ = 0.821	*k* = −8→6
5249 measured reflections	*l* = −20→18

### Refinement

**Table:**

Refinement on *F*^2^	Primary atom site location: structure-invariant direct methods
Least-squares matrix: full	Secondary atom site location: difference Fourier map
*R*[*F*^2^ > 2σ(*F*^2^)] = 0.036	Hydrogen site location: inferred from neighbouring sites
*wR*(*F*^2^) = 0.094	H atoms treated by a mixture of independent and constrained refinement
*S* = 1.04	*w* = 1/[σ^2^(*F*_o_^2^) + (0.0429*P*)^2^ + 0.678*P*] where *P* = (*F*_o_^2^ + 2*F*_c_^2^)/3
2451 reflections	(Δ/σ)_max_ = 0.001
194 parameters	Δρ_max_ = 0.46 e Å^−^^3^
1 restraint	Δρ_min_ = −0.46 e Å^−^^3^

### Special details

**Table:**

Geometry. All esds (except the esd in the dihedral angle between two l.s. planes) are estimated using the full covariance matrix. The cell esds are taken into account individually in the estimation of esds in distances, angles and torsion angles; correlations between esds in cell parameters are only used when they are defined by crystal symmetry. An approximate (isotropic) treatment of cell esds is used for estimating esds involving l.s. planes.
Refinement. Refinement of F^2^ against ALL reflections. The weighted R-factor wR and goodness of fit S are based on F^2^, conventional R-factors R are based on F, with F set to zero for negative F^2^. The threshold expression of F^2^ > 2sigma(F^2^) is used only for calculating R-factors(gt) etc. and is not relevant to the choice of reflections for refinement. R-factors based on F^2^ are statistically about twice as large as those based on F, and R- factors based on ALL data will be even larger.

### Fractional atomic coordinates and isotropic or equivalent isotropic displacement parameters (Å^2^)

**Table:**

	*x*	*y*	*z*	*U*_iso_*/*U*_eq_	
Br1	0.68536 (3)	0.08870 (6)	0.13010 (2)	0.05792 (18)	
Cl1	0.20872 (10)	0.35802 (17)	0.20909 (7)	0.0738 (4)	
N1	0.2386 (2)	0.2805 (4)	0.03030 (15)	0.0362 (6)	
N2	0.1252 (2)	0.3513 (4)	0.02797 (16)	0.0405 (7)	
O1	0.4312 (2)	0.3783 (4)	−0.14532 (14)	0.0534 (7)	
O2	−0.05797 (18)	0.3567 (3)	0.07125 (14)	0.0533 (7)	
C1	0.4308 (2)	0.2898 (4)	−0.02015 (18)	0.0358 (7)	
C2	0.4949 (3)	0.3117 (5)	−0.08532 (19)	0.0402 (8)	
C3	0.6132 (3)	0.2662 (5)	−0.0855 (2)	0.0484 (9)	
H3	0.6552	0.2811	−0.1287	0.058*	
C4	0.6700 (3)	0.1984 (5)	−0.0217 (2)	0.0468 (9)	
H4	0.7496	0.1668	−0.0221	0.056*	
C5	0.6074 (3)	0.1783 (5)	0.04254 (19)	0.0401 (8)	
C6	0.4885 (3)	0.2236 (4)	0.04331 (19)	0.0390 (8)	
H6	0.4472	0.2093	0.0868	0.047*	
C7	0.3058 (3)	0.3422 (4)	−0.01972 (19)	0.0375 (8)	
H7	0.2752	0.4208	−0.0560	0.045*	
C8	0.0425 (2)	0.2909 (4)	0.07350 (18)	0.0362 (7)	
C9	0.0717 (2)	0.1349 (4)	0.12478 (18)	0.0348 (8)	
C10	0.1435 (3)	0.1509 (5)	0.1879 (2)	0.0445 (9)	
C11	0.1611 (3)	0.0055 (7)	0.2367 (2)	0.0578 (11)	
H11	0.2078	0.0194	0.2799	0.069*	
C12	0.1087 (4)	−0.1580 (6)	0.2202 (3)	0.0627 (12)	
H12	0.1215	−0.2560	0.2521	0.075*	
C13	0.0377 (4)	−0.1789 (6)	0.1573 (3)	0.0613 (11)	
H13	0.0037	−0.2910	0.1465	0.074*	
C14	0.0166 (3)	−0.0332 (5)	0.1098 (2)	0.0476 (9)	
H14	−0.0338	−0.0464	0.0682	0.057*	
C15	0.4912 (4)	0.4085 (6)	−0.2124 (2)	0.0649 (12)	
H15A	0.5509	0.4995	−0.2042	0.097*	
H15B	0.4357	0.4488	−0.2505	0.097*	
H15C	0.5276	0.2977	−0.2278	0.097*	
H2	0.109 (4)	0.444 (4)	−0.0024 (19)	0.080*	

### Atomic displacement parameters (Å^2^)

**Table:**

	*U*^11^	*U*^22^	*U*^33^	*U*^12^	*U*^13^	*U*^23^
Br1	0.0437 (2)	0.0720 (3)	0.0575 (3)	0.00185 (18)	−0.00739 (17)	0.0012 (2)
Cl1	0.0748 (7)	0.0728 (8)	0.0728 (8)	−0.0254 (6)	−0.0124 (6)	−0.0101 (6)
N1	0.0305 (12)	0.0354 (16)	0.0430 (17)	0.0034 (11)	0.0047 (11)	0.0041 (13)
N2	0.0293 (13)	0.0413 (17)	0.051 (2)	0.0057 (11)	0.0055 (12)	0.0133 (14)
O1	0.0489 (14)	0.0648 (18)	0.0473 (16)	0.0000 (12)	0.0109 (12)	0.0140 (13)
O2	0.0318 (12)	0.0558 (16)	0.0728 (19)	0.0101 (11)	0.0087 (11)	0.0244 (14)
C1	0.0330 (15)	0.0330 (18)	0.042 (2)	−0.0017 (13)	0.0054 (14)	−0.0016 (15)
C2	0.0435 (18)	0.035 (2)	0.042 (2)	−0.0066 (15)	0.0062 (15)	−0.0031 (16)
C3	0.0410 (18)	0.051 (2)	0.054 (2)	−0.0043 (16)	0.0197 (17)	−0.0017 (19)
C4	0.0323 (16)	0.048 (2)	0.061 (3)	−0.0004 (15)	0.0104 (16)	−0.0026 (19)
C5	0.0356 (16)	0.0345 (19)	0.050 (2)	−0.0040 (14)	0.0011 (15)	−0.0035 (16)
C6	0.0347 (16)	0.0371 (19)	0.046 (2)	−0.0060 (14)	0.0086 (15)	−0.0037 (16)
C7	0.0392 (17)	0.0310 (18)	0.042 (2)	0.0000 (13)	0.0045 (15)	0.0010 (15)
C8	0.0312 (15)	0.0343 (19)	0.043 (2)	−0.0012 (13)	−0.0012 (13)	0.0042 (15)
C9	0.0285 (15)	0.0348 (19)	0.042 (2)	0.0046 (12)	0.0089 (14)	0.0051 (15)
C10	0.0339 (16)	0.050 (2)	0.049 (2)	0.0010 (15)	0.0013 (16)	−0.0007 (18)
C11	0.047 (2)	0.075 (3)	0.051 (3)	0.014 (2)	0.0002 (17)	0.017 (2)
C12	0.056 (2)	0.062 (3)	0.071 (3)	0.012 (2)	0.012 (2)	0.031 (2)
C13	0.067 (3)	0.039 (2)	0.079 (3)	−0.0035 (19)	0.017 (2)	0.010 (2)
C14	0.0476 (19)	0.044 (2)	0.051 (2)	−0.0011 (16)	0.0098 (17)	0.0049 (18)
C15	0.066 (2)	0.079 (3)	0.050 (3)	−0.011 (2)	0.014 (2)	0.012 (2)

### Geometric parameters (Å, °)

**Table:**

Br1—C5	1.897 (3)	C5—C6	1.386 (4)
Cl1—C10	1.733 (4)	C6—H6	0.9300
N1—C7	1.282 (4)	C7—H7	0.9300
N1—N2	1.384 (3)	C8—C9	1.504 (4)
N2—C8	1.340 (4)	C9—C10	1.377 (5)
N2—H2	0.893 (10)	C9—C14	1.409 (5)
O1—C2	1.368 (4)	C10—C11	1.395 (5)
O1—C15	1.422 (4)	C11—C12	1.371 (6)
O2—C8	1.235 (3)	C11—H11	0.9300
C1—C6	1.384 (5)	C12—C13	1.373 (6)
C1—C2	1.408 (4)	C12—H12	0.9300
C1—C7	1.466 (4)	C13—C14	1.388 (5)
C2—C3	1.380 (5)	C13—H13	0.9300
C3—C4	1.390 (5)	C14—H14	0.9300
C3—H3	0.9300	C15—H15A	0.9600
C4—C5	1.384 (5)	C15—H15B	0.9600
C4—H4	0.9300	C15—H15C	0.9600
			
C7—N1—N2	114.6 (3)	O2—C8—C9	120.2 (3)
C8—N2—N1	121.5 (3)	N2—C8—C9	119.0 (3)
C8—N2—H2	120 (3)	C10—C9—C14	118.5 (3)
N1—N2—H2	119 (3)	C10—C9—C8	123.4 (3)
C2—O1—C15	118.1 (3)	C14—C9—C8	118.0 (3)
C6—C1—C2	119.0 (3)	C9—C10—C11	121.3 (4)
C6—C1—C7	121.1 (3)	C9—C10—Cl1	119.4 (3)
C2—C1—C7	119.9 (3)	C11—C10—Cl1	119.2 (3)
O1—C2—C3	124.7 (3)	C12—C11—C10	119.3 (4)
O1—C2—C1	115.2 (3)	C12—C11—H11	120.4
C3—C2—C1	120.1 (3)	C10—C11—H11	120.4
C2—C3—C4	120.4 (3)	C11—C12—C13	120.8 (4)
C2—C3—H3	119.8	C11—C12—H12	119.6
C4—C3—H3	119.8	C13—C12—H12	119.6
C5—C4—C3	119.5 (3)	C12—C13—C14	120.2 (4)
C5—C4—H4	120.2	C12—C13—H13	119.9
C3—C4—H4	120.2	C14—C13—H13	119.9
C4—C5—C6	120.5 (3)	C13—C14—C9	119.9 (4)
C4—C5—Br1	119.5 (2)	C13—C14—H14	120.1
C6—C5—Br1	120.0 (3)	C9—C14—H14	120.1
C1—C6—C5	120.5 (3)	O1—C15—H15A	109.5
C1—C6—H6	119.8	O1—C15—H15B	109.5
C5—C6—H6	119.8	H15A—C15—H15B	109.5
N1—C7—C1	120.4 (3)	O1—C15—H15C	109.5
N1—C7—H7	119.8	H15A—C15—H15C	109.5
C1—C7—H7	119.8	H15B—C15—H15C	109.5
O2—C8—N2	120.7 (3)		

### Hydrogen-bond geometry (Å, °)

**Table:**

*D*—H···*A*	*D*—H	H···*A*	*D*···*A*	*D*—H···*A*
N2—H2···O2^i^	0.89 (1)	1.99 (1)	2.882 (4)	175 (4)

Symmetry codes: (i) −*x*, −*y*+1, −*z*.
